# Trophic State Drives the Diversity of Protists in a Tropical River (New River, Belize)

**DOI:** 10.3390/microorganisms10122425

**Published:** 2022-12-07

**Authors:** Maximiliano Barbosa, Forrest W. Lefler, David E. Berthold, Venetia S. Briggs-Gonzalez, Frank J. Mazzotti, H. Dail Laughinghouse

**Affiliations:** 1 Agronomy Department, Ft. Lauderdale Research and Education Center, University of Florida/IFAS, 3205 College Avenue, Davie, FL 33314, USA; 2 Wildlife Ecology and Conservation Department, Ft. Lauderdale Research and Education Center, University of Florida/IFAS, 3205 College Avenue, Davie, FL 33314, USA

**Keywords:** protist, eutrophication, phytoplankton, tropical, metabarcoding, taxonomic and functional diversity

## Abstract

Land use disrupts the ecosystem functioning of freshwater systems and significantly affects trophic state. Consequently, biodiversity is severely affected by changes to the ecosystem. Microbial eukaryotes (i.e., protists) play an essential role in ecosystem functioning, contributing to biogeochemical processes, nutrient cycling, and food webs. Protist composition is a useful biological quality parameter for monitoring aquatic ecosystems and determining aquatic system health. In this study, we investigated the effects of land usage and trophic state on the communities of microbial eukaryotes in the New River (Belize, C.A.). Land use and trophic state both significantly affected protist community compositions, with impacted and mesotrophic sampled sites having higher biodiversity when compared to other sites. Autotrophic organisms dominated indirectly impacted and eutrophic sites, while impacted and mesotrophic sites had proportional ratios of autotrophic and heterotrophic organisms. Our study highlights the significant effects of trophic gradients on protistan community composition, even at the local scales.

## 1. Introduction

Freshwater ecosystems contribute disproportionately to global biological richness, harboring about 6% of all described species [1], while only covering 0.8% of the Earth’s surface [2]. In addition, freshwater ecosystems provide vital resources for humans, including economic productivity (e.g., fisheries) and provision of ecosystem services (e.g., cleaning water) [3]. Freshwater habitats—and the biodiversity they support—are especially vulnerable to land use change. Over the last century, human exploitation of freshwater resources has risen steeply, leading to growing threats to biodiversity around the world [1]. Specifically, rivers are highly impacted by anthropogenic activity. Over 50% of freshwater runoff is trapped by humans, through use of dams, and over 25% of global sediment loads are trapped in reservoirs before they reach the ocean [4,5]. Land use change has resulted in excessive loading of nutrients and pollution to lakes and rivers causing eutrophication, which has resulted in the inability of these habitats to support their natural biotic communities. Consequently, human disturbance has negatively affected species diversity and endemism in highly impacted geographic regions [6]. 

Species richness and composition is strongly correlated to ecosystem function. However, the extent of the effect of biodiversity loss on ecosystem function is largely dependent on the identity of the species being lost and the ecological process under consideration [6]. Microorganisms account for most of the biodiversity in freshwater ecosystems and are major components of freshwater aquatic ecosystems [7,8]. Specifically, cyanobacteria (blue-green algae), microbial eukaryotes (protists), and bacteria dominate the diversity in freshwater ecosystems and include important groups of primary producers that can transform nutrients along food webs in aquatic ecosystems and heterotrophic organisms that can decompose organic matter [9]. 

Protists make up much of the eukaryotic diversity, live in virtually all environments, and their biomass at global scales is immense [10,11]. However, protists have received less attention than their bacterial counterparts in all areas of research of environmental microbiology [12]. In addition, studies exploring the diversity of freshwater protists are scarce compared to studies in oceans [13]. Thus, there is still much to discover regarding protistan diversity, their influencing factors, and distribution patterns, especially in subtropical and tropical environments. Protistan community composition is affected by interspecies interactions, physiochemical conditions, and seasonal changes [14]. Due to their sensitivity to water quality changes or increased water pollution, the protistan community composition, particularly primary producers (phytoplankton), is a useful biological quality parameter for monitoring aquatic ecosystems and determining aquatic system health [15].

High throughput DNA sequencing (HTS) has become commonplace in surveying microbial community structure, making it feasible to characterize microbial diversity at multiple levels of resolution by obtaining hundreds of thousands of DNA sequences from environmental samples [16]. However, HTS investigations on microorganisms in freshwater habitats usually focus on bacteria and frequently overlook photosynthetic, heterotrophic, and mixotrophic protists. Though there is substantial work on marine protists [17,18,19,20,21], only a few studies have evaluated these communities in fresh waters, and those freshwater studies have largely focused on temperate and alpine environments [22,23,24,25]. Applications of HTS to freshwater samples have unveiled a large breadth of diversity in microbial eukaryotic communities and enabled investigation of evolutionary dynamics in microbial assemblages and their ecological interactions [16]. For example, metabarcoding studies have revealed the prevalence of parasitic and other symbiotic relationships across protistan lineages in aquatic and terrestrial environments [26]. Therefore, HTS technologies offer a reliable method of microbial community evaluations. 

The New River in Belize is an example of a hotspot for biodiversity and a highly impacted river within a tropical environment [27]. In 2019, the New River system in the northern Belizean lowlands experienced a historic dry season, which resulted in the accumulation of nutrients and pollutants in the river system for a long period of time. These factors led to the development of algal blooms and anaerobic conditions throughout the river system. We conducted a study aimed at describing and investigating relationships between environmental conditions, land usage, trophic state and protistan community structure along a spatial scale in the New River. We hypothesized that land use and the trophic state of the river would significantly affect the protistan biodiversity and that protistan communities would change along a latitudinal gradient along the river. Here, we present the changes in protistan community structure along a trophic state gradient from New River Lagoon to Corozal Bay, which encompasses much of the New River system.

## 2. Materials and Methods

### 2.1. Study Area

**Figure 1:**
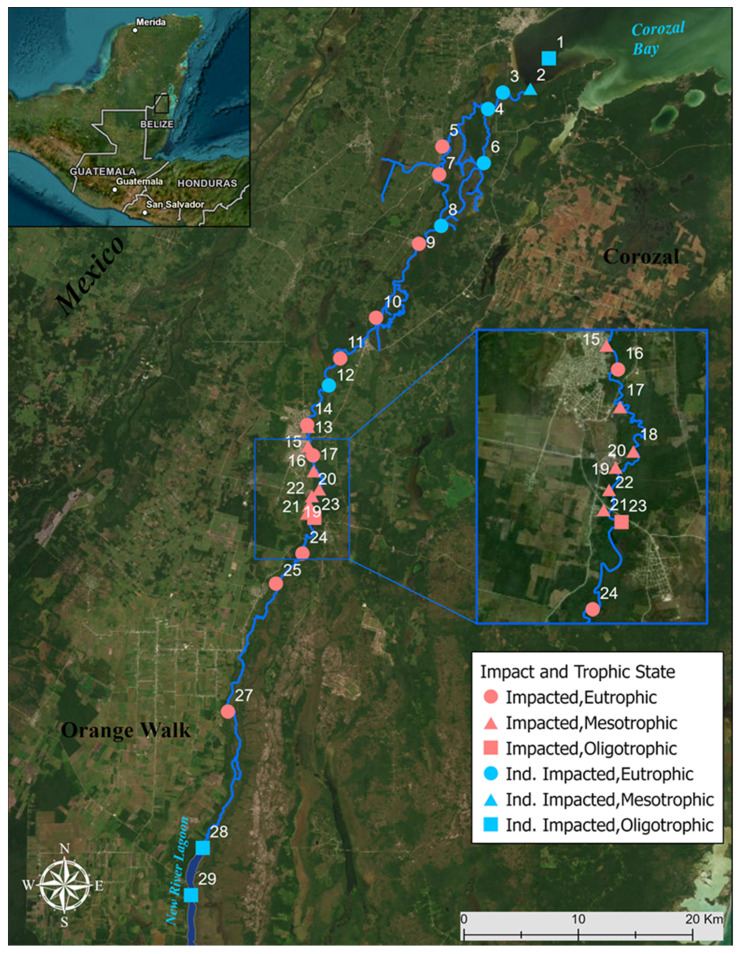
Map of 29 sample sites, with site 26 removed, along the New River (Blue line). New River runs through three Belize districts (Orange Walk, Belize, and Corozal) from New River Lagoon to Corozal Bay. Legend demonstrates trophic state and impact of sampled sites.

The New River (Belize, C.A.) was sampled along its length beginning at the New River Lagoon (17°44′31.5″ N 88°38′59.8″ W) in the south, toward the middle of New River (18°05′33.5″ N 88°33′28.4″ W) near Orange Walk town and ending at Corozal Bay (18°22′01.1″ N 88°22′07.8″ W) in the north. Twenty-nine samples were collected between 5–10 October 2019 at approximately 10 km intervals (Figure 1). To make comparison easier, sites were divided into 3 regions—Southern region (sites 24–29), middle of the river (sites 10–23), and northern region (sites 1–9). Regions were based on proximity to New River Lagoon (Southern), Orange Walk town (Middle) and Corozal Bay (Northern).

### 2.2. Sampling Methods

Water samples were collected using 1 L Nalgene bottles for use in environmental DNA extractions. Additional water samples were collected using 50 mL falcon tubes for nutrient analysis and trace element analysis. Samples for trace element analysis were acidified with nitric acid and stored at room temperature. Sample filters for environmental DNA and water samples for nutrient analyses were frozen and sent to University of Florida Fort Lauderdale Research and Education Center to be processed the same week of collection. In the field, water quality measurements—dissolved oxygen, water temperature, pH, salinity, conductivity, turbidity, chlorophyll-a concentration, and phycocyanin concentration—were taken using a YSI EXO3 (Xylem Inc., Washington, DC, USA) multiparameter sonde on site. Depth was measured using a Secchi disk (Fisher Scientific, Waltham, MA, USA) and was used to estimate water transparency and calculate photic depth (Zeu) (Table S1).

### 2.3. Laboratory Methods

Immediately after arriving in the laboratory, water samples stored in Nalgene bottles were filtered through a 0.45 µm glass filter (MilliporeSigma, Burlington, MA, USA) and stored at −20 °C for amplicon sequencing. Additional water samples were frozen and stored for nutrient composition analysis. Total phosphorus and total nitrogen were determined using the persulfate digestion method [28] and analyzed using a Seal AutoAnalyzer (Seal AA500; Seal Analytical, WI, USA). Biologically relevant elements (Boron, Copper, Calcium, Potassium, Sodium, Iron, Cobalt, Magnesium, Manganese, Aluminum, and Zinc) were quantified using an Avio 200 ICP-OES (Inductively Coupled Plasma Optical Emission Spectrometer) (PerkinElmer, Waltham, MA, USA) following Standard Method 3120 [29].

### 2.4. Trophic State Index

The multivariate index of trophic state TRIX [30] was used to characterize the trophic state of the New River system and Corozal Bay. The TRIX index is based on dissolved oxygen saturation (DO), chlorophyll-a (Chl-a), Total Nitrogen (TN), and Total Phosphorus (TP) concentrations and was calculated according to the equation TRIX = log (Chl-a × DO (%) × TN × TP) − (−1.5)/1.2. The trophic state was classified based on the threshold values proposed by Caruso et al. [31], ranging from Excellent: <2 (ultra-oligotrophic); High: 2 < TRIX < 4 (oligotrophic); Good: 4 ≤ TRIX ≤ 5 (Mesotrophic); Moderate: 5 ≤ TRIX ≤ 6 (Mesotrophic to Eutrophic); and Poor: 6 ≤ TRIX < 8 (Eutrophic).

### 2.5. Anthropogenic Impact Classification

The occurrence of urban, industrial, and agricultural development along the New River system and Corozal Bay was used to classify the sampled locations as anthropogenically (i.e., directly) impacted or indirectly impacted. Due to the prevalence of non-point source pollution in the river system, sampled sites were considered indirectly impacted if there were no human settlements adjacent to the riparian zone or stream channel, or obvious signs of water discharge within 100 m of the site. Potentially impaired sites were considered impacted if agricultural, urban, industrial, or aquacultural developments were present within 100 m of the riparian zone. Specifically, impacted zones often showed obvious signs of disturbances, such as wastewater treatment infrastructure, treatment ponds, wastewater discharge, sewage pipes, floating trash, and motorized aerators installed by the Belizean Department of Environment (Figure S1). In summary, sampling sites were categorized on anthropogenic impact, which was based on observed human developments along the river (e.g., industry, urban centers, and agriculture), and validated by the Department of Environment of Belize based on human land use adjacent to the river.

### 2.6. DNA Extraction and Amplification

DNA was extracted from filters using a DNeasy Blood and Tissue Kit (Qiagen, Hilden, Germany), modified according to Djurhuus et al. [32]. Fragments (~530 bp) encompassing the V4 region of the 18S rRNA gene were amplified using EK-565F-NGS (5′-GCAGTTAAAAAGCTCGTAGT-3′) and UNonMet (5′-TTTAAGTTTCAGCCTTGCG-3′), the latter biased against metazoans [33]. Primers were tagged using specific 10-bp molecular identifiers (MIDs) for multiplexed sequencing. To minimize PCR-associated biases, triplicate PCR reaction products were pooled per sample. PCR reactions were conducted in a 25 µL reaction mixture containing 5 µL of eluted DNA (~10 µg), 0.75 µL F primer (0.2 µM), 0.75 µL R primer (0.2 µM), 6 µL of PCR grade water, and 12.5 µL of 1× KAPA HiFi HotStart ReadyMix (Roche Molecular Systems, Pleasanton, CA, USA) for 35 cycles (94 °C for 30 s, 55–58 °C for 30–45 s, 72 °C for 90 s) preceded by 2 min denaturation at 94 °C and followed by 5 min extension at 72 °C. Pooled amplicons were purified using AMPure XP beads (Beckman Coulter Life Sciences, Brea, CA, USA) following manufacturer protocol.

### 2.7. Library Preparation

Amplicon libraries were indexed using Nextera V2 indexing kit (Illumina, Inc., San Diego, CA, USA) following manufacturer protocols [34]. Indexed amplicon products were purified using AMPure XP beads following manufacturer protocol. Purified amplicon products were then normalized to 4 ± 1 nM and quantified using Qubit 2.0 Fluorometer (Invitrogen, Waltham, MA, USA). Normalized amplicon products were sequenced using paired-end (2 × 300 bp) Illumina MiSeq at the Interdisciplinary Center for Biotechnology Research (ICBR) (University of Florida, Gainesville, FL, USA).

### 2.8. Sequence and Phylogenetic Analysis

Amplicon sequences were demultiplexed and assigned to specific sample IDs based on their MIDs at ICBR using an in-house bioinformatic pipeline. R software (4.2) and Dada2 (1.26) were used to process raw sequences [35,36]. Paired-end reads were filtered, trimmed, and merged under strict criteria. Cleaned and merged reads were dereplicated and subsequently analyzed for detection and removal of potential chimeras using Dada2. Non-chimeric sequences were pooled together to define amplicon sequence variants (ASVs). Taxonomic assignment of ASVs was based on naïve Bayesian classifying method and the 18S rRNA database PR2 v4.13 was used as reference [37]. An ASV table with the read abundance was generated for diversity and statistical analyses [38]. Non-protistan (e.g., non-protistan Opisthokonts and Embryophyta) ASVs were removed prior to downstream analysis. Phylogenetic analyses were conducted to further explore the overall diversity of the most diverse supergroups generated within our ASV table. Sequences that were not assigned a taxonomic rank lower than “supergroup” were aligned to full 18S rRNA reference sequences from PR2 database, covering the eukaryotic diversity, using MAFFT and uninformative sites were removed using trimAI [39,40]. A phylogenetic tree was built using full reference sequences with IQ-tree under a GTR + I + G4 sequence evolutionary model which was determined using ModelTestNG [41,42]. 

### 2.9. Statistical Analysis

Sequence read abundances were filtered and normalized to the median sampling depth using phyloseq package in R [43]. ASVs present in less than 5% of samples were filtered to avoid low abundance taxa, and samples with less than 1000 reads were excluded from subsequent analysis. Site 26 was discarded from further analyses as no ASVs belonging to protistan phyla were generated. The vegan package was used for statistical analyses, generation of rarefaction curves, calculation of richness and diversity indices, and generation of ordinations [44]. Evenness was calculated using the microbiome package [45]. Prior to analyses, the data were transformed using Hellinger method to avoid biases toward rare species and minimize influence of most abundant groups. Similarities in protist communities among locations, trophic state, and anthropogenic impact were explored using the Non-Metric Multidimensional Scaling (NMDS) analysis with Bray–Curtis dissimilarities. In addition, a redundancy analysis (RDA) was employed to find significant relationships between environmental variables and protist diversity. Using the “adonis2” function of the vegan package, a permutational multivariate analysis of variance (PERMANOVA) was conducted using Bray–Curtis dissimilarities to test the effect of trophic state and anthropogenic impact on protist community composition. Lastly, a similarity percentages (SIMPER) analysis was employed to identify the protistan group driving dissimilarities among trophic states and impacted locations. 

### 2.10. Protist Functional Diversity Analysis

We evaluated the distribution of major protist functional groups following methods by Singer et al. [46]. In summary, generated ASVs were classified within three major functional groups based on their taxonomic affiliations according to the three main trophic modes: autotrophs, heterotrophs, and parasites. The autotrophic group was comprised of obligate phototrophs (e.g., Chlorellales) and mixotrophs (e.g., Cryptomonadales), heterotrophic group was comprised of phagotrophic predators (e.g., Colpodellida and Hypotrichia). The parasitic group was comprised of Oomycota and Perkinsida (Table S2). A one-way analysis of variance (ANOVA) was conducted to determine if relative abundance of functional groups was significantly different.

## 3. Results

### 3.1. Water Quality Parameters

Water quality assessments demonstrated low levels of dissolved oxygen (DO) along the river. DO levels were typically hypoxic (<3 mg/L) with some locations having anoxic levels (<0.5 mg/L). The salinity along the river ranged from fresh (0.29 ppt) to brackish (30.25 ppt) at sites near Corozal Bay. A spatial difference of chlorophyll a concentration was observed in the water column. Chlorophyll a concentrations were low in New River Lagoon and Corozal Bay (1.14 μg/L ≤ Chl a ≤ 1.60 μg/L) and were high, although highly variable, across New River (6.71 μg/L ≤ Chl a ≤ 60.5 μg/L). Nitrogen concentrations were lower than 100 μg/L across the sampling locations and were only detected at concentrations higher than 100 μg/L in sites 6, 7, 11, 12, and 24. Variable concentrations of phosphorus were detected across sampling sites. Sites 7, 9, and 10 showed phosphorus concentrations higher than 100 μg/L, with site 10 reaching the highest phosphorus concentration of 1039 μg/L (Table S1).

### 3.2. Trophic State Indices and Anthropogenic Impact

The New River system covers a wide range of trophic conditions according to the TRIX values. Most of New River was characterized as eutrophic with 15 out of 28 sites in the river having eutrophic conditions. In addition, nine sites in New River had mesotrophic conditions while four sites had oligotrophic conditions—two sites in New River Lagoon, one in Corozal Bay, and one site in New River. In addition, a total of 20 sites were considered anthropogenically impacted due to large presence of human disturbance while only 9 sites were considered indirectly impacted (Table S1).

### 3.3. Protist Community Composition

A total of 95,402 high-quality reads were generated after low-quality reads were discarded which were subsequently clustered into 1199 unique ASVs (Table S1). After removing non-protistan ASVs, a total of 1073 were used for downstream analysis. Taxonomy was assigned to the lowest level possible, but for the purpose of comparative analysis of protist communities, we chose a taxonomic cut-off at the order level as it accurately represented the overall protist community composition. Some group designations contain an “X” to indicate that this group level does not have an order name according to the database used for taxonomic classification (e.g., “Prostomatea 1_X”). ASV richness, based on rarefaction curves, and Shannon and Simpson indices, was highest in sites to the south of the river and in Corozal bay, and lowest toward the north of the river (Figures S2 and S3). Evenness, based on Pielou index, was also highest in sites toward to the south of the river and Corozal Bay and lowest toward to the south of the river (Table S1). 

**Figure 2:**
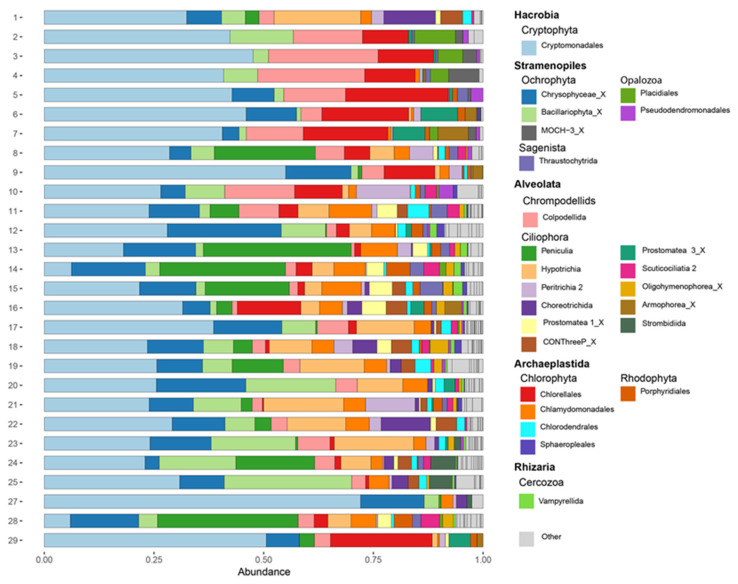
Relative abundance of protists, at the order level, within sampling sites, during 5–10 October 2019, along the New River, Belize. The 25 most abundant protistan groups are included.

Phylogenetic analyses showed high diversity of protists, specifically in the supergroups Alveolata, Archaeplastida, and Stramenopila. Phylogenetic trees demonstrated ASVs were present in most of the major clades of Alveolata and Stramenopila, and a large number of ASVs were present in 2 clades of Archaeplastida (Figures S4–S6). Similarly, relative read abundance showed that all the protistan groups were highly diverse and highly variable in relative abundance along the New River system (Figure 2). 

Taxonomic assignment using Bayesian classifying method generated ASVs affiliated with 16 phyla belonging to 6 supergroups (Hacrobia, Alveolata, Stramenopila, Archaeplastida, Rhizaria, and Apusozoa). Protist communities were dominated by Cryptomonadales, Chrysophyceae_X, Bacillariophyta_X, and Peniculia, representing 32.3%, 10.5%, 7.4%, and 7.0% relative sequence abundance, respectively. Colpodellida, Chlorellales, and Hypotrichia represented 6.9%, 6.6%, and 5.9% of relative sequence abundance, respectively, and other groups were detected at lower proportions. Based on relative read abundance, the Cryptomonadales was the most abundant group in the New River system, including New River Lagoon and Corozal Bay (Figure 2). The second and third most abundant groups were Chrysophyceae_X and Bacillariophyta_X which were also present throughout the New River system but at lower abundances compared to Cryptomonadales. The fourth and fifth most abundant groups were Peniculia and Colpodellida which were also abundant throughout the New River system, but Peniculia was found mostly in the middle of the river and in New River Lagoon, and Colpodellida was most abundant in brackish waters near the mouth of the river. The sixth most abundant group was the Chlorellales, which was most abundant near the mouth of the river, in one site in the middle of New River, and in New River Lagoon. The seventh and eight most abundant groups were Hypotrichia and Chlamydomonadales and most abundant in the middle of New River. The rest of the groups were most abundant in the middle of New River; however, some groups were present at considerable abundances in Corozal Bay and New River Lagoon such as Choreotrichida and Prostomatea 1_X, respectively. 

**Figure 3:**
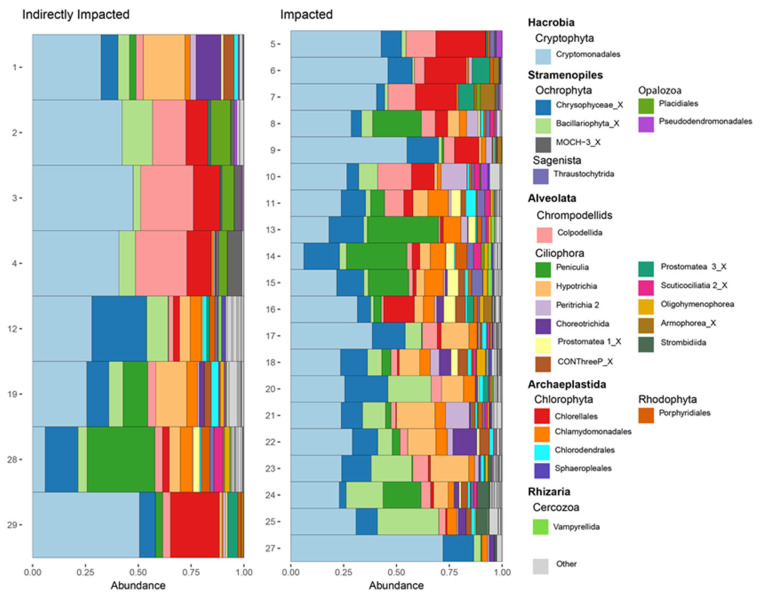
Relative abundance of protists, at the order level, within sampling sites, during 5–10 October of 2019, along the New River, Belize based on trophic state. The 25 most abundant protistan groups are included.

**Figure 4:**
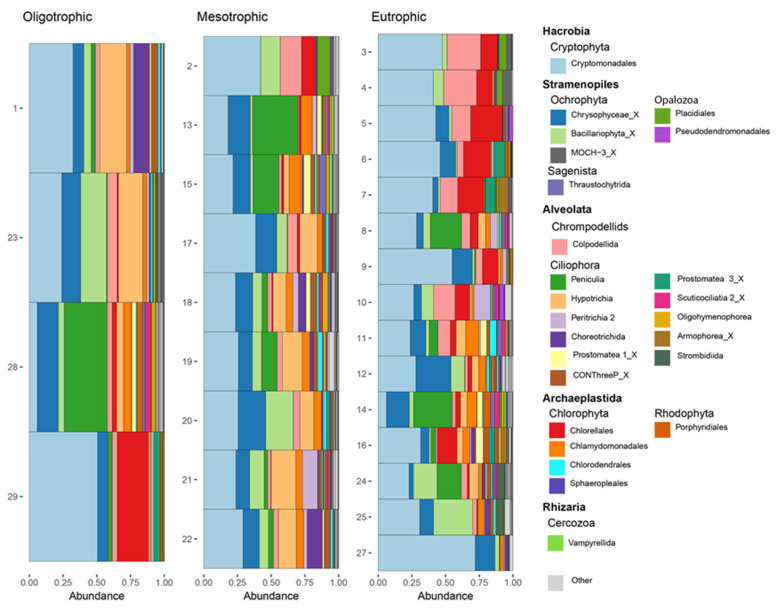
Relative abundance of protists, at the order level, within sampling sites, during 5–10 October 2019, along the New River, Belize based on impacted status from October 2019. The 25 most abundant protistan groups are included.

Relative read abundance based on trophic state was very similar between oligotrophic, mesotrophic, and eutrophic sites (Figure 3). Cryptomonadales, Chrysophyceae_X, Bacillariophyta_X, and Peniculia dominated overall abundance regardless of trophic state. However, Chlorellales were most abundant in eutrophic sites with some considerable presence in one mesotrophic site and one oligotrophic site. Hypotrichia were present at considerable read abundance in oligotrophic and mesotrophic sites, but very seldomly in eutrophic sites. Chlamydomonadales were present within all trophic states, but at low read abundances. Impacted and indirectly impacted sites had very similar protistan group distributions based on relative abundance (Figure 4).

**Figure 5:**
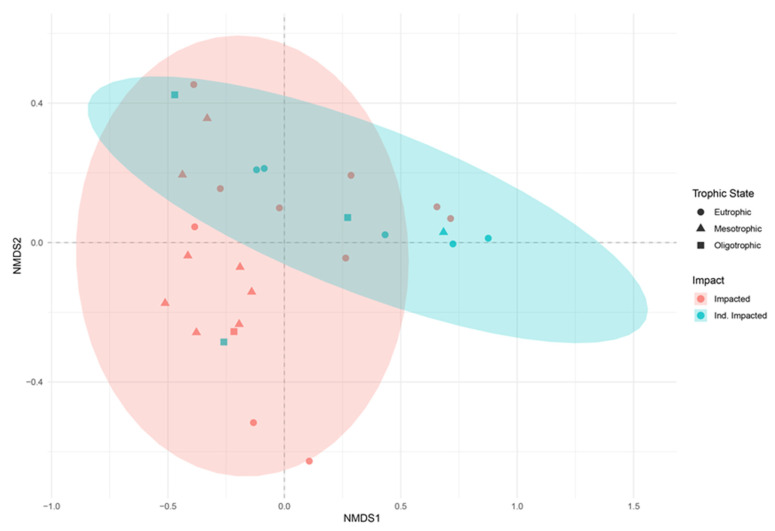
Non-metric Multidimensional Scaling ordination, within two dimensions of protists communities within sampling sites, during 5–10 October 2019, along the New River, Belize. Legend shows trophic state and impact status. Ellipses represent the location of group centroids to at a 0.95 confidence level. Stress level: 0.07.

**Figure 6:**
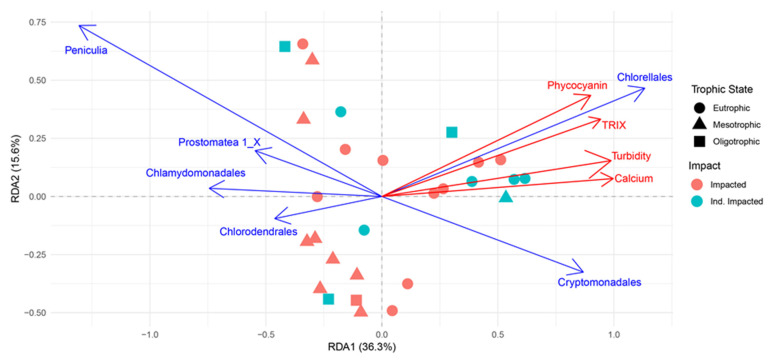
Redundancy analysis ordination, within two dimensions of protists communities within sampling sites, during 5–10 October 2019, along the New River, Belize. Legend shows trophic state and impact status. Significant taxonomic drivers of variation are shown by blue arrows. Significant environmental drivers are shown by red arrows.

The non-metric multidimensional scaling (NMDS) analysis showed a large overlap between protist communities based on trophic state but did show a marked differentiation among sites based on impact (Figure 5; Stress: 0.07). The redundancy analysis (RDA) showed variance was significantly driven by trophic state (TRIX), total phosphorus (TP), phycocyanin, chlorophyll a, turbidity, and calcium concentrations and 6 groups (Peniculia, Protomatea 1_X, Chlorodendrales, Cryptomonadales, Chlorellales, Chlamydomonadales) were driving 65% of variance and dispersion based on RDA analyses (Figure 6). The PERMANOVA showed that there was a significant effect on protist communities by anthropogenic impact (*p* < 0.05) and trophic state (*p* < 0.05). A pairwise PERMANOVA demonstrated that protist communities in mesotrophic locations were significantly different than protist communities in eutrophic locations. SIMPER analyses demonstrated 20 groups contributed to 71% of the dissimilarity between impacted and indirectly impacted sites, and 20 groups contributed to 71% of the dissimilarity between mesotrophic and eutrophic sites. Specifically, five groups contributed significantly to the dissimilarities between impacted and indirectly impacted sites (Table 1), and two groups contributed significantly to the dissimilarities between mesotrophic and eutrophic sites (Table 2).

### 3.4. Spatial Distribution of Functional Groups

**Figure 7:**
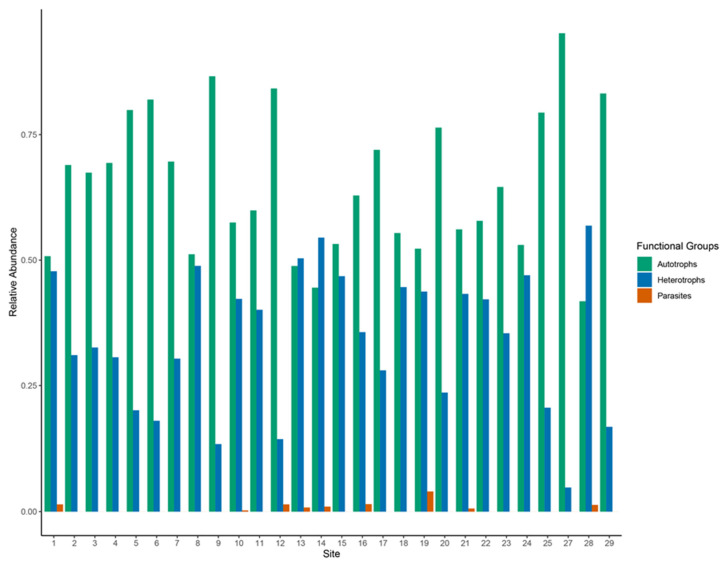
Relative abundance bar plot of functional groups in protists communities per sampling sites, during 5–10 October 2019, along the New River, Belize. Colors indicate functional groups—green = Autotrophs; blue = Heterotrophs; red = Parasites.

**Figure 8:**
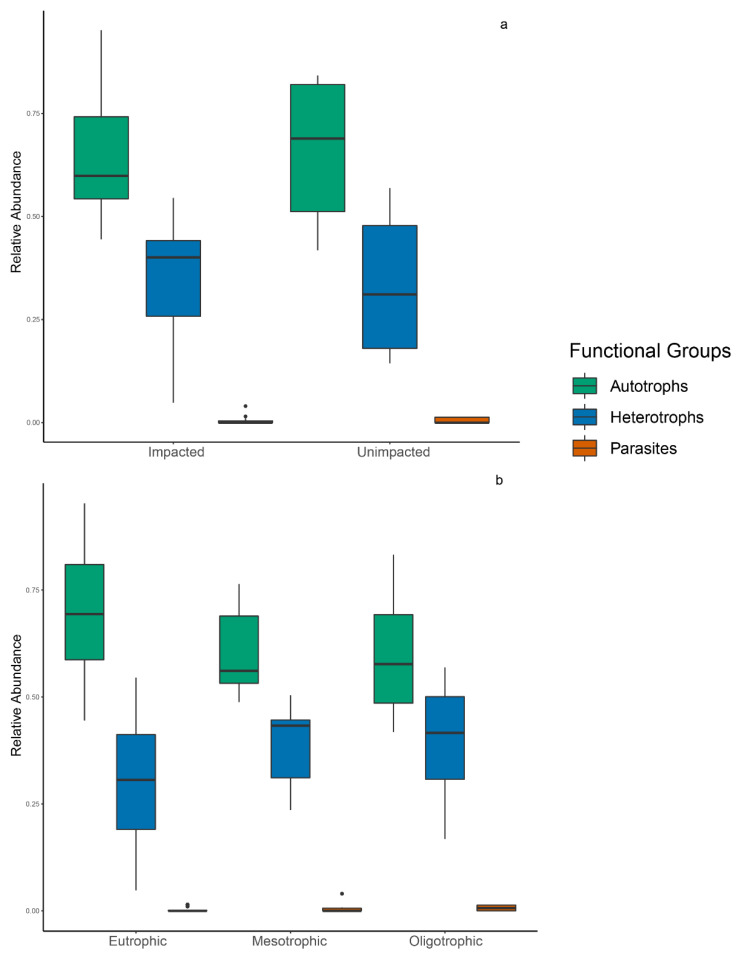
Relative abundance box plot of functional groups in protists communities within impact status (**a**) and trophic state (**b**), during 5–10 October 2019, along the New River, Belize. Colors indicate functional groups—green = Autotrophs; blue = Heterotrophs; red = Parasites.

Relative abundance of the autotrophs, heterotrophs, and parasitic groups was highly variable among sites (Figure 7). The autotrophic functional group was largely dominant throughout the river system and heterotrophs having a lesser but considerable abundance throughout the river. Parasites had very low abundance throughout the river system. The relative abundance of functional groups was significantly different based on trophic state (*p* ≤ 0.05). The autotrophic functional group was dominant in all trophic states and was also highest in eutrophic sites (Figure 8). In addition, the eutrophic state had considerably lower abundance of heterotrophic functional groups than autotrophic functional groups. Autotrophic functional groups were higher than heterotrophic functional groups in both mesotrophic and oligotrophic sites. Parasitic functional groups were present in all trophic states but were more abundant in the mesotrophic sites. The relative abundance of functional groups was not significantly different based on anthropogenic impact. Impacted and indirectly impacted sites were both dominated by autotrophic functional groups with heterotrophic groups considerably lower in abundance (Figure 8). The parasitic functional group was only present in impacted sites at a considerable abundance.

## 4. Discussion

The New River is a largely understudied tropical river in Belize and there are few records on its historical water quality which makes comparisons on the change of water quality and impacts over time difficult. Our data are thus pioneering and fundamental for future studies in this region. The New River flows along several river towns and communities and is used as a source of drinking water, recreation, transport, and fishing. There is extensive land use around much of New River and we found significant evidence of these impacts on water quality. Much of New River is surrounded by human developments, such as urban, agriculture, industrial, and aquacultural industries which are often linked to disturbances of river water quality. Hypoxic conditions were pervasive throughout the river with a frequent occurrence of microalgal blooms composed mainly of picocyanobacteria (pers. obs.). In agreement, primary productivity based on chlorophyll a, phycocyanin, TN, and TP was high throughout the river. Discharge from domestic sewage, industrial wastewater, and runoff from agricultural fertilizers and pesticides are common pollution sources for many freshwater ecosystems [47,48,49,50]. According to the trophic state index (TRIX), most of the river was eutrophic or mesotrophic, with only four sites being oligotrophic. Although not all sites that were considered impacted had eutrophic conditions, most sites considered indirectly impacted had either mesotrophic or eutrophic conditions. This suggests that nonpoint source pollution (i.e., nutrients, pesticides, fertilizer, and organic and inorganic matter) has an indirect impact on parts of the river that are considered indirectly impacted. Nonpoint source pollution can be carried through water by surface and subsurface runoff from rain events and by irrigation return flows [51]. River communities are greatly affected by water quality, and increased water pollution negatively affects biodiversity of aquatic ecosystems [52]. In our study, water quality or increased water pollution was strongly correlated with decreased protist biodiversity. 

The trophic state of water bodies has previously been associated with significant differences in biodiversity between aquatic ecosystems based on trophic state. Lee and Liu [53] found that mesotrophic freshwater lakes are often more biodiverse than oligotrophic or eutrophic lakes. In addition, Lefranc et al. [54] and Zhao et al. [55] found that the population biodiversity of microbial protists was higher in mesotrophic lakes than in oligotrophic lakes. There are few published studies relating trophic state of rivers to overall protist biodiversity and even fewer in tropical rivers, but our study provides novel data that indicates that like freshwater lakes, mesotrophic conditions correlate to higher biodiversity of protists in rivers. 

Relative read abundance results indicate that protistan communities were variable along New River and were most differentiated among trophic state and impacted/indirectly impacted sites. In addition, statistical analyses show that trophic state and anthropogenic impact significantly affected the protist community assemblages. Moreover, the protistan functional groups differed significantly based on trophic state. Autotrophic functional groups were prevalent in all trophic states, but they were especially dominant in eutrophic environments. These results also indicate the extent of the effects of human impact on the protist community composition. Land usage (i.e., agricultural development and industrialization) can impact the environmental conditions of aquatic ecosystems through the discharge of pollutants and nutrients which alter community composition and structure [56]. Santos et al. [57] found that human land use significantly affects soil protist biodiversity and community composition. In addition, Zou et al. [58] found that anthropogenic activities significantly affect aquatic protist biodiversity in a highly impacted urban ecosystem. Our study confirms that land use significantly affects protist biodiversity and community composition. In addition, our study also demonstrates that the degree of impact to aquatic ecosystems, based on trophic state, is an important factor for protistan biodiversity. 

The NMDS showed that protist communities were significantly differentiated based on impacted and indirectly impacted sites. In addition, SIMPER analyses demonstrated that protistan communities were highly dissimilar between impacted and indirectly impacted sites, and between mesotrophic and eutrophic sites. This suggests that anthropogenic impact and trophic state significantly affect the overall protist assemblages in tropical river ecosystems. The RDA showed that environmental variables significantly affected the protist communities of New River. Specifically, phycocyanin and chlorophyll *a* concentrations and TRIX were significantly associated with Chlorellales and Chlorodendrales. In addition, turbidity and calcium concentrations were negatively associated with Chlamydomonadales and Chlorodendrales. Our results suggest that Chlorellales is successful in eutrophic states while Chlorodendrales is more successful in oligotrophic states, low turbidity, and low calcium concentrations. Moreover, Chlamydomonadales is more successful in low turbidity and low calcium concentrations. Chlorellales, Chlorodendrales, and Chlamydomonadales are groups of photoautotrophic protists within the green algae which compete for resources such as light, carbon dioxide, and essential nutrients to reproduce, however, our results suggest that these groups are more or less successful depending on the trophic state of their habitat. In addition, the positive and negative associations with phycocyanin concentrations indicate that cyanobacteria may be a potential driver of protistan diversity. This possible driver is currently being assessed by a supplemental study in the New River, Belize. 

Inferring relative abundances of individual groups based on sequence data is a known shortcoming in using eDNA metabarcoding of protistan communities [59]. However, assessing ecosystem functionality from sequence data results in highly similar assessments as those obtained from morphospecies data and experiments have shown that abundance-based indices, such as Bray–Curtis index, can successfully mirror protistan mock communities [60,61]. Therefore, we are confident that the conclusions drawn from our study are well supported by previous experimental evidence. Although our study is lacking temporal data, due to complications in the collection of additional data in Belize (COVID pandemic) and the unavailability of historical data in the New River system, our spatial data supports the occurrence of discrete protistan communities along the river. Moreover, our study highlights the significant differences in protistan biodiversity along a spatial scale in a highly impacted riverine system. Both anthropogenic impact and trophic state significantly affect diversity and protistan community assemblages. Spatial trends indicate that even local diversity of protists can be highly variable at small scales.

## 5. Conclusions

The present study demonstrates the high variability in protist communities in a highly impacted river system. The New River experiences extensive non-point source pollution due to the prevalent land use surrounding the river. Anthropogenic pollution considerably affects the water chemistry of the river which in turn affects the protistan communities of the river. Our data highlights the significant differences between protists communities experiencing direct and indirect impact. In addition, our study shows that trophic state is a significant driver of protist communities and that the communities in mesotrophic environments are more diverse compared to those in oligotrophic and eutrophic environments. Lastly, our study demonstrated that protistan functional groups are significantly different according to the trophic state of the environment they inhabit. Specifically, autotrophic organisms appear to be dominant in all environments, but were even more dominant in eutrophic environments. These results are highly indicative of the influence of eutrophication on the prevalence of autotrophic organisms.

## Figures and Tables

**Table 1:** Similarity percentage analysis to identify protist orders that best differentiated impacted and indirectly impacted sites during 5–10 October 2019, along the New River, Belize. Contribution percentage for each group and cumulative percentage (up to 70%) are shown. Mean relative abundance of protist groups is shown for impacted and indirectly impacted sites. Groups with significant contributions to dissimilarities are in bold.

Protist Group	Contribution%	Cumulative%	Impacted Sites Abundance	Indirectly Impacted Sites Abundance
Peniculia	6.00	6.00	7.04	6.86
Chlorellales	6.00	13.00	5.09	9.94
**Chrysophyceae_X**	6.00	18.00	11.61	8.14
Hypotrichia	5.00	24.00	0.02	0.04
Cryptomonadales	5.00	28.00	30.68	35.83
Colpodellida	4.00	33.00	5.45	9.96
Bacillariophyta_X	4.00	37.00	8.12	5.76
**Chlamydomonadales**	4.00	41.00	4.58	2.12
Choreotrichida	4.00	44.00	1.88	1.31
**Placidiales**	3.00	48.00	0.24	2.21
Prostomatea_3_X	3.00	51.00	0.78	1.79
Peritrichia_2	3.00	53.00	2.38	1.37
CONThreeP_X	3.00	56.00	1.64	0.58
Prostomatea_1_X	3.00	59.00	1.58	0.73
**Chlorodendrales**	3.00	61.00	1.45	0.67
**MOCH.3_X**	2.00	64.00	0.13	1.35
Porphyridiales	2.00	66.00	0.96	1.25
Scuticociliatia_2	2.00	69.00	0.96	0.74
Thraustochytrida	2.00	71.00	1.12	0.78

**Table 2:** Similarity percentage analysis to identify protist groups that best differentiated sites with mesotrophic and eutrophic trophic states during 5–10 October 2019, along the New River, Belize. Contribution percentage for each group and cumulative percentage (up to 70%) are shown. Average relative abundance of protist groups is shown for mesotrophic and eutrophic sites. Groups with significant contributions to dissimilarities are in bold.

Protist Group	Contribution%	Cumulative%	Mesotrophic Sites Abundance	Eutrophic Sites Abundance
Peniculia	6.00	6.00	8.37	5.41
Chlorellales	6.00	13.00	1.82	9.49
**Hypotrichia**	6.00	19.00	9.27	2.46
Chrysophyceae_X	5.00	24.00	12.27	9.24
Colpodellida	4.00	28.00	4.81	8.78
Bacillariophyta_X	4.00	32.00	8.65	6.62
Cryptomonadales	4.00	36.00	27.61	36.25
Choreotrichida	4.00	40.00	2.59	0.77
Chlamydomonadales	3.00	43.00	5.27	3.15
Peritrichia_2	3.00	46.00	2.55	1.87
CONThreeP_X	3.00	49.00	2.01	0.88
Prostomatea_1_X	3.00	52.00	1.64	1.11
Placidiales	3.00	55.00	1.11	0.91
Thraustochytrida	2.00	57.00	0.99	1.17
Chlorodendrales	2.00	60.00	1.64	0.97
Prostomatea_3_X	2.00	62.00	0.45	1.43
**Oligohymenophorea_X**	2.00	64.00	1.28	0.40
Scuticociliatia_2	2.00	67.00	0.85	0.83
Porphyridiales	2.00	69.00	0.71	1.16
MOCH.3_X	2.00	71.00	0.19	0.86

## Data Availability

The raw reads were deposited into the NCBI Sequence Read Archive (SRA) database (BioProject: PRJNA849233).

## Supplementary Materials

The following supporting information can be downloaded at: https://www.mdpi.com/article/10.3390/microorganisms10122425/s1, Figure S1: Human disturbance occurring along New River, Belize; Figure S2: Rarefaction analysis of ASVs; Figure S3: Alpha Diversity. Legend shows trophic state and impact status; Figure S4: Phylogenetic tree and classification of Alveolata ASVs; Figure S5: Phylogenetic tree and classification of Archaeplastida ASVs; Figure S6: Phylogenetic tree and classification of Stramenopiles ASVs. Table S1: Sampling sites in New River, Belize and associated physico-chemical and diversity data; Table S2: Tentative classification of phylogenetic lineages within broad functional categories.
