# Supplementary material for: Trophic State Drives the Diversity of Protists in a Tropical River (New River, Belize)
Source: Microorganisms. 2022 Dec 7;10(12):2425. doi: 10.3390/microorganisms10122425 (PMC9782246; doi:10.3390/microorganisms10122425)
Supplement: Supplementary file 1 [file microorganisms-10-02425-s001.zip › microorganisms-2068403-Supplementary Figures.pdf]

microorganisms

Article

Trophic State Drives the Diversity of Protists in a Tropical River (New River, Belize)

Maximiliano Barbosa [1], Forrest W. Lefler [1], David E. Berthold [1], Venetia S. Briggs-Gonzalez [2], Frank J. Mazzotti [2] and H. Dail Laughinghouse IV [1,*]

[1] Agronomy Department, Ft. Lauderdale Research and Education Center, University of Florida/IFAS, 3205 College Avenue, Davie, FL 33314, USA
[2] Wildlife Ecology and Conservation Department, Ft. Lauderdale Research and Education Center, University of Florida/IFAS, 3205 College Avenue, Davie, FL 33314, USA
* Correspondence: hlaughinghouse@ufl.edu

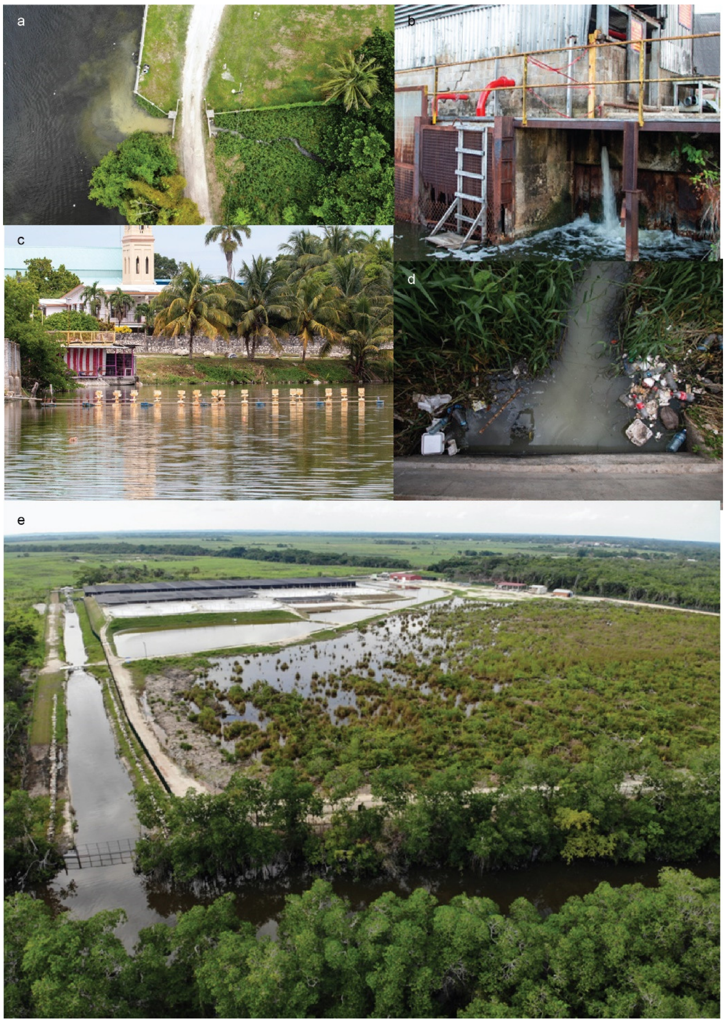

Figure S1. Human disturbance occurring along New River, Belize. (**a**) Aerial image of waste discharge into New River; (**b**) Wastewater discharge into New River; (**c**) Motorized aerator installed in New River by the Department of Environment; (**d**) Close up of refuse and potential pollutants flowing into New River; (**e**) Aerial image of man-made lagoon and treatment ponds. Photos by Justin Dalaba.

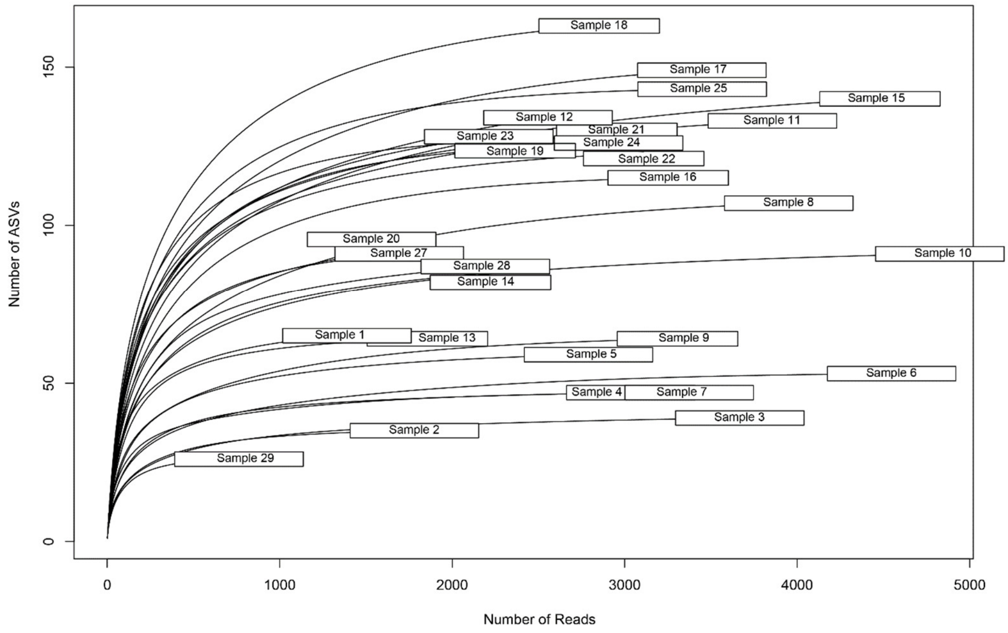

Figure S2. Rarefaction analysis of ASVs.

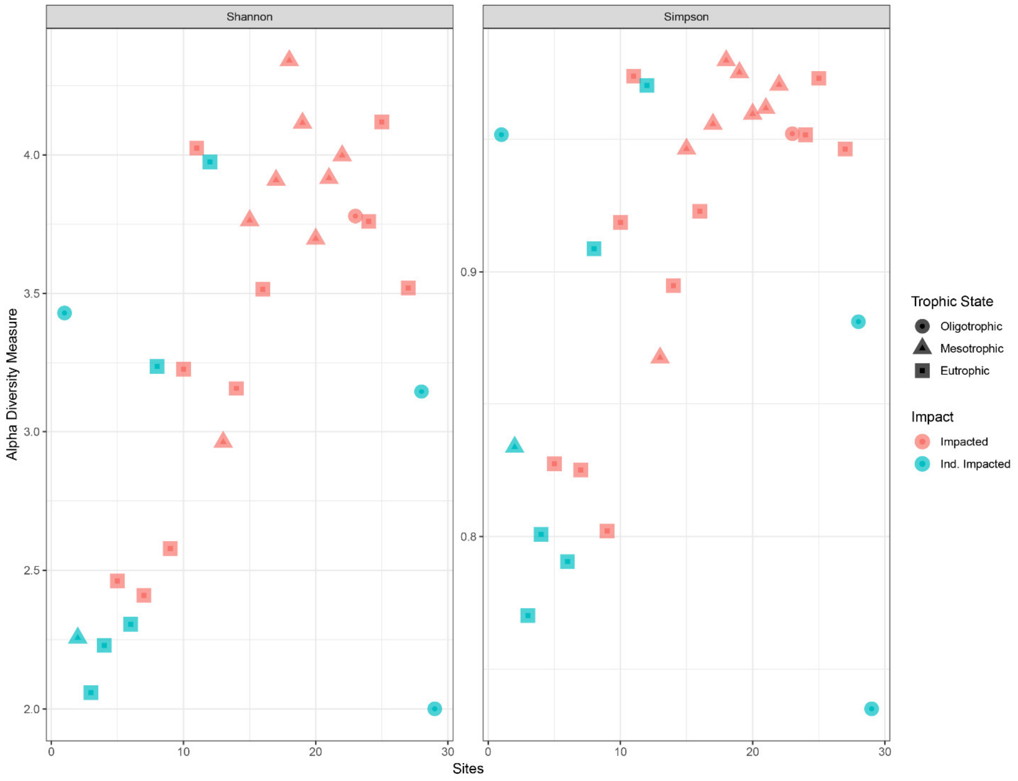

Figure S3. Alpha Diversity. Legend shows trophic state and impact status. Different shapes of points are based on trophic levels – square = Eutrophic; triangle = Mesotrophic; circle = Oligotrophic. Color of points indicate impact – directly impacted = red; indirectly impacted = blue. X axis indicates site and y axis indicates alpha diversity measure.

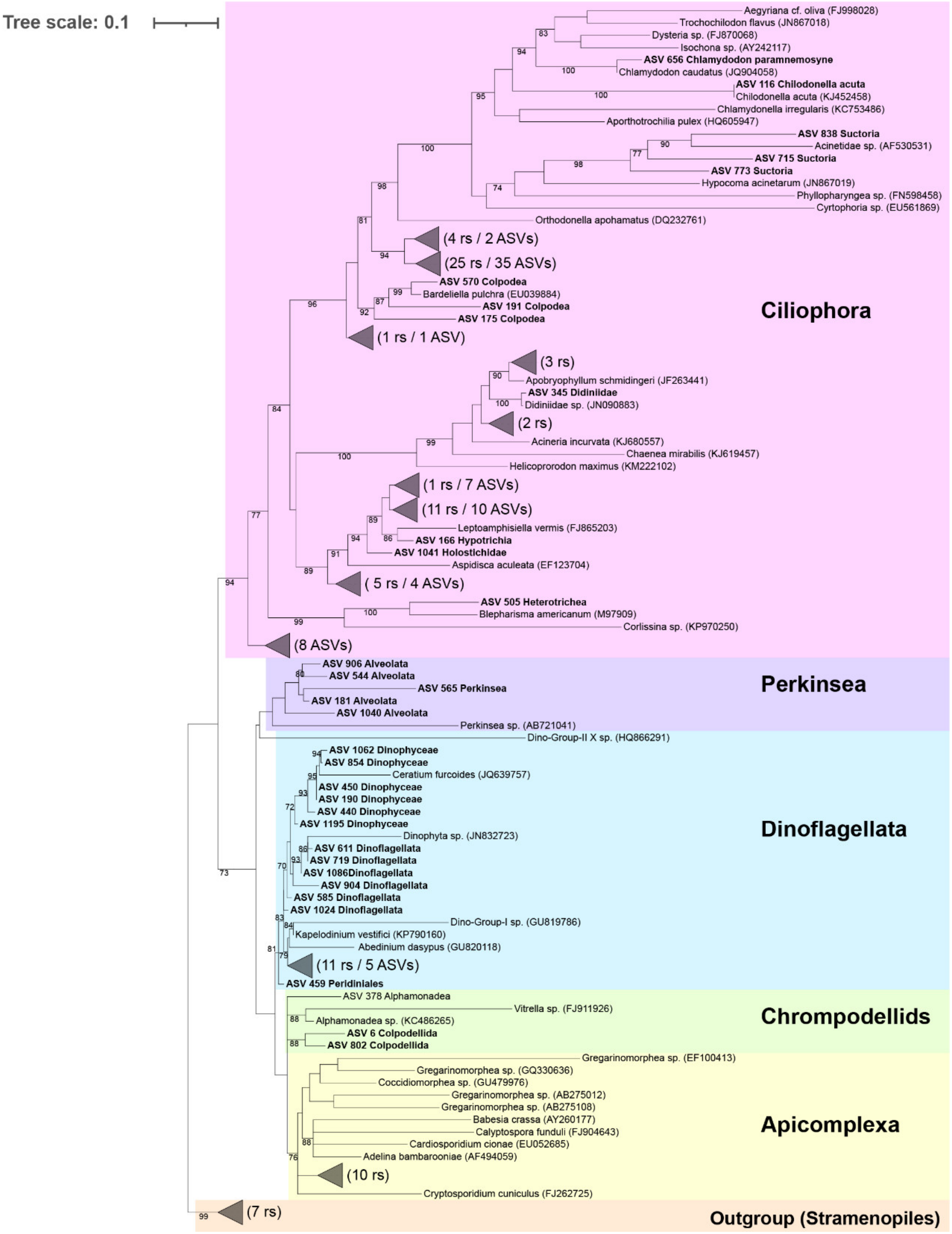

Figure S4. Phylogenetic tree and classification of Alveolata ASVs. Maximum likelihood bootstrap support ≥ 65 are shown at nodes. Collapsed clusters were annotated with number of reference sequences (rs) followed by the number of ASVs. The tree is rooted on Stramenopiles, a closely related supergroup.

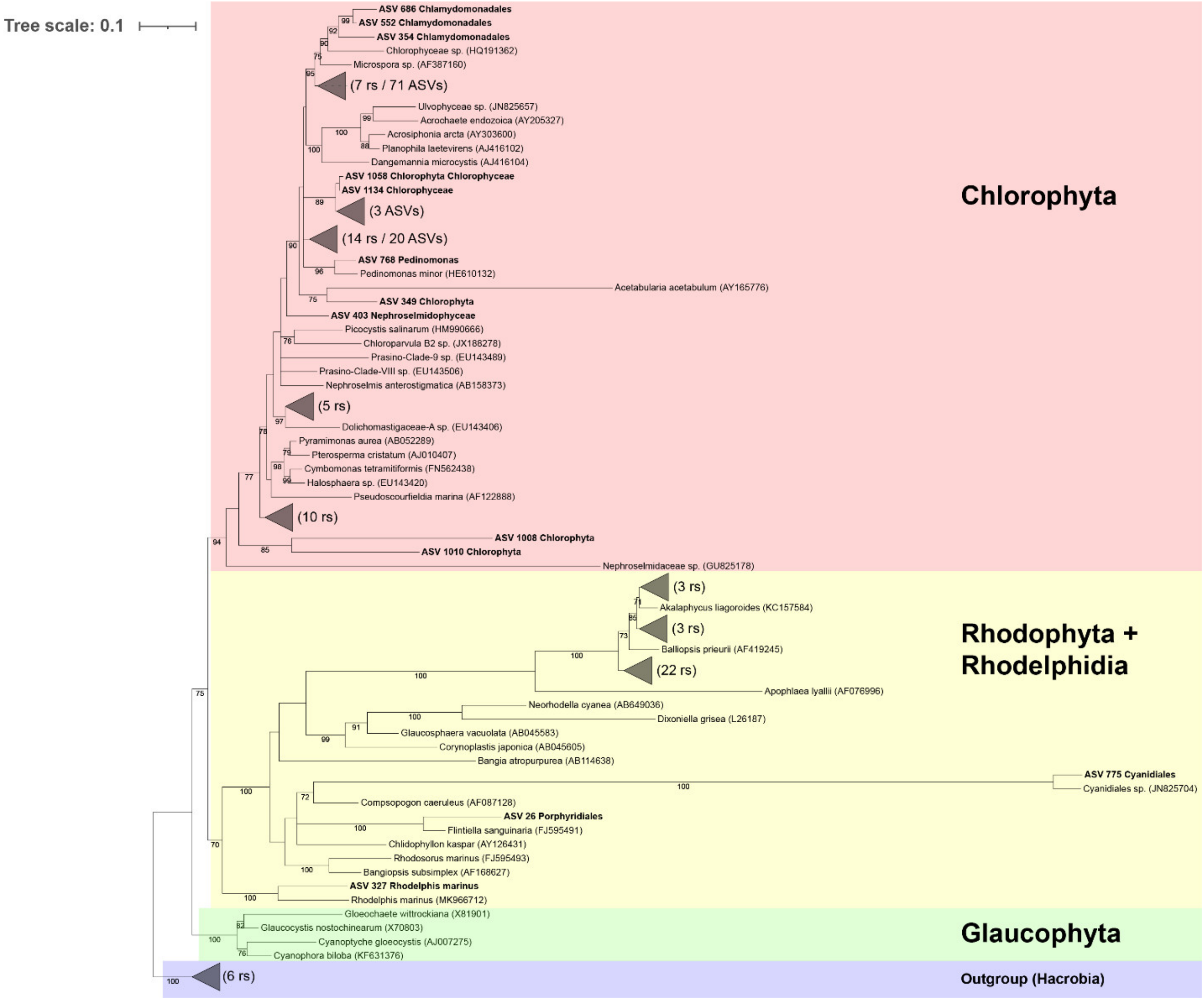

Figure S5. Phylogenetic tree and classification of Archaeplastida ASVs. Maximum likelihood bootstrap support ≥ 65 are shown at nodes. Collapsed clusters were annotated with number of reference sequences (rs) followed by the number of ASVs. The tree is rooted on Hacrobia, a closely related supergroup.

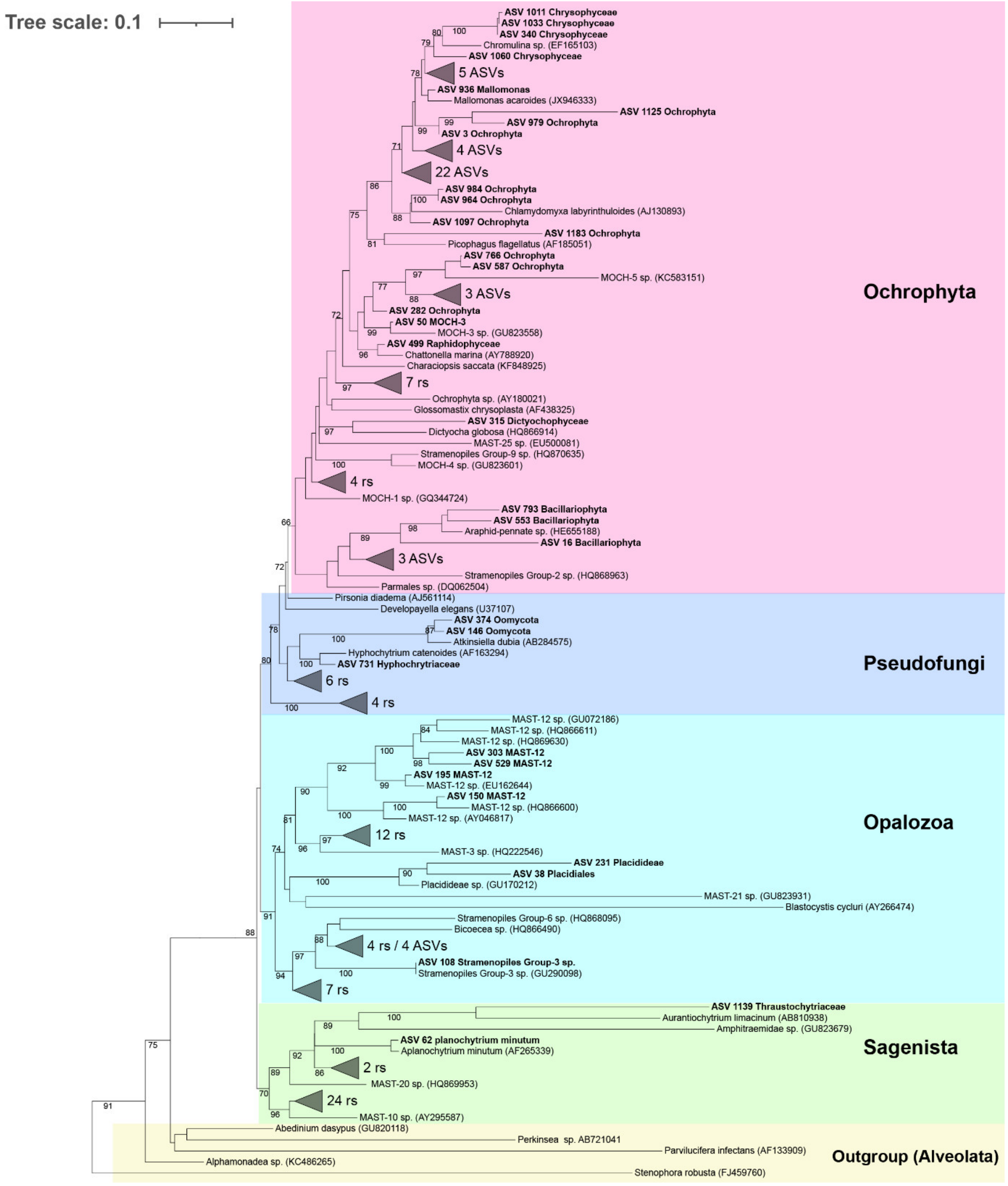

Figure S6. Phylogenetic tree and classification of Stramenopiles ASVs. Maximum likelihood bootstrap support ≥ 65 are shown at nodes. Collapsed clusters were annotated with number of reference sequences (rs) followed by the number of ASVs. The tree is rooted on Alveolata, a closely related supergroup.
